# On the Origins of Symbiotic Fungi in Carmine Cochineals and Their Function in the Digestion of Plant Polysaccharides

**DOI:** 10.3390/insects15100783

**Published:** 2024-10-09

**Authors:** Pilar González-Román, Diana Hernández-Oaxaca, Rafael Bustamante-Brito, Marco A. Rogel, Esperanza Martínez-Romero

**Affiliations:** Center for Genomic Sciences, Universidad Nacional Autónoma de México, UNAM Universidad SN, Cuernavaca 62210, Morelos, Mexico

**Keywords:** insect symbiosis, carminic acid, dimorphic fungi, *Periconia*, *Aspergillus*, holobiont

## Abstract

**Simple Summary:**

The domesticated carmine cochineal produces pigments used in food, cosmetics, and the textile industry. Its use dates to the pre-Hispanic period and thus is an Amerindian legacy. Cochineals grow and feed on the edible leaves of cacti. Since insects feed on plant sap, they may obtain their microbes from the plants and indeed we found here that some of the fungi in the insect guts are the same as those encountered in cacti. Fungi may help in digestion as they are capable of breaking down plant polymers in cultures and inside guts. We found fungi inside cochineals during microscopic examinations using different procedures to detect fungi. One of the isolated fungi produces a purple pigment that resembles the carminic acid. This study identified several potential functions of cochineal fungi and examined their plant origin.

**Abstract:**

The cochineal insect *Dactylopius coccus* Costa (Hemiptera) has cultural and economic value because it produces carminic acid that is used commercially. In this study, distinct fungi were cultured from dissected tissue and identified as *Penicillium*, *Coniochaeta*, *Arthrinium*, *Cladosporium*, *Microascus*, *Aspergillus*, and *Periconia*. Fungi were microscopically observed inside cochineals in the gut, fat body, and ovaries. Since cochineals spend their lives attached to cactus leaves and use the sap as feed, they can obtain fungi from cacti plants. Indeed, we obtained *Penicillium*, *Aspergillus*, and *Cladosporium* fungi from cacti that were identical to those inside cochineals, supporting their plant origin. Fungi could be responsible for the degrading activities in the insect guts, since cellulase, pectinase, and amylase enzymatic activities in insect guts decreased in fungicide-treated cochineals. Our findings set the basis for the further study of the interactions between insects, fungi, and their host plants.

## 1. Introduction

Fungi and insects are two of the most diverse groups of eukaryotes [1] and in insects, fungi may have a vast diversity of effects from obligate mutualisms to parasitism [2,3]. These associations have been studied in several insect groups such as ants [4], wasps [5], termites [6], leafhoppers [7], bees [8], and beetles [9]. The environmental success of insects has been linked to their beneficial associations with symbiotic microbes. Fungal symbionts may provide insects with vitamins, sterols, or enzymes to obtain sugars from plant polysaccharides [10,11]. Most hemipterans are plant-sucking insects; thus, with nutritional deficiencies in their diet, these insects would be prone to a dependency on microbial symbionts [12].

Fungal symbionts from some scale insects are known [13,14]. A comprehensive review of fungi in the soft scale insects from Hemiptera has been published, showing the fungi present in different species, their location within insects, and their possible transmission to offspring [15]. In many cases, fungi reside in the fat body or hemolymph and have thick cell walls. It is remarkable that fungi have substituted bacterial symbionts in a few cases. Seemingly, in Hemiptera, fungal symbioses are less common in Sternorrhyncha compared to Auchenorrhyncha [15]. Seven Coccidae species from the Mediterranean area [16] and 28 species from Southern China [17], which feed on plant sap, contained members of the *Ophiocordyceps*-allied fungi. The presence of *Ophiocordyceps* in different developmental stages of soft scale insects was explored, finding that it predominated in the first and second instar nymphs [17]. *Ophiocordyceps* are considered the primary symbionts because they were found in all tested insects and in eggs [16]. In contrast, yeast-like symbionts were observed in the cytoplasm of fat body cells but not in eggs of *Kermes quercus* from the coccoid family Kermesidae [18]. However, in the mango mealybug *Rastrococcus iceryoides* (Coccoidea: Pseudococcidae), a transovarial transmission of yeast-like symbionts was suggested [18].

The carmine cochineal insect *Dactylopius coccus* (Costa, 1835) is a hemipteran that belongs to the superfamily Coccoidea and the family Dactylopiidae. It originated in Oaxaca, Mexico [13] and feeds on *Opuntia* cacti, mostly *Opuntia ficus-indica* (Mill, 1768), to which females remain attached for most of their life cycle. *D. coccus* shows extreme sexual dimorphism; adult males are several times smaller in size, winged, and have a short lifespan of only a few days. *D. coccus* is one of the few domesticated insects in Mexico and has been cultivated since pre-Hispanic times because of its carminic acid, a red pigment that changes color depending on pH. Carmine is used in the cosmetic, food, and textile industries [19]. In 2019, Bustamante-Brito et al. [20] found evidence of nitrogen fixation in *D. coccus* by the betaproteobacteria *Candidatus* Dactylopiibacterium carminicum, which may provide vitamins and amino acids to the insect. *Spiroplasma* and *Wolbachia* may be found as well inside cochineals [21,22].

In the Dactylopiidae family, *Cryptococcus* has been isolated from *D. opuntiae* (Cockerell, 1896) *D. confusus*, and *D. coccus*, whereas *Rhodotorula* has been isolated from *D. confusus* and *D. coccus*, *Trametes* from *D. opuntiae*, *Debaryomyces* from *D. confusus*, and *Phanerochaete*, *Irpex*, *Stereum*, *Periconia*, and *Penicillium* have only been described in *D. coccus* [23]. The functional roles of most fungal symbionts from scale insects remain unknown. A previous report on a fungal community associated with *D. coccus* described the role of *Cryptococcus* in uric acid catabolism in the cochineal [22]. The microbial digestion of plant polysaccharides is required since insects lack the enzymes to hydrolyze the cellulose, hemicellulose, pectin, and lignin commonly found in plant diets [24,25]. *Penicillium*, *Cladosporium*, and *Aspergillus* are known to be capable of producing pectinases, cellulases, and xylanases [26]. In termites that feed on wood, fungi contribute to plant polysaccharide degradation [27], but in Coccoidea, specifically in Dactylopiidae, it is not known how complex sugars are catabolized.

The resident microorganisms of herbivores may have a plant [28] or a maternal origin [29]. This work aimed to define the possible origins of fungal symbionts in the cochineals and to explore some of the functional roles of fungal symbionts in *D. coccus*.

## 2. Materials and Methods

### 2.1. Sampling

Insects were donated by a farm in Tepoztlan, Morelos (18.990441, −99.117425) that harvests cochineals for carmine production. Only adult females were used in this study. Insects were sampled with the cacti they were attached to since they cannot survive without them.

### 2.2. Fungal Isolation from Dactylopius coccus Tissues and Cultures

For fungal isolation, 30 adult females were detached from different cacti and rinsed with 100% and 70% ethanol and sterile water twice for 1 min each to remove the external wax and dust. They were then dissected under sterile conditions; the cuticle was removed, and internal organs (whole gut, ovaries, and fat body) were placed in 1 mL of PBS buffer. Immediately, the tissue was homogenized in a mortar, then inoculated in PDA (Potato Dextrose Agar: 200 g of potato per liter, 20 g dextrose, 20 g agar), ME (Malt Extract: 20 g malt extract and 20 g agar), PY (Peptone Yeast: 20 g peptone, 10 g dextrose, 10 g yeast extract, and 20 g agar), and YPD (Yeast Peptone Dextrose: 20 g dextrose, 10 g peptone, 5 g yeast extract, and 20 g agar) culture plates at three dilutions (10^0^, 10^−1^, and 10^−2^). Petri dishes were inoculated using beads and incubated at 28 °C for 22 days. Fungal colonies were subcultured in the corresponding medium and then stored in a liquid medium with 30% glycerol at −70 °C.

We tested different growth conditions for *Coniochaeta* sp: two oxygen concentrations (microaerobic and aerobic); YPD and a medium with 40% filtered insect extract; and three different agar concentrations (0.5%, 1%, and 1.5%) at two different temperatures (28 °C and 37 °C). We incubated for 72 h and then applied lactophenol blue to a sample from each condition to visualize the predominant cellular type with a Leica microscope with LAS DFC295 software, at 20 and 40×.

### 2.3. Light and Transmission Electron Microscopy (TEM) of D. coccus Tissue

Dissected insect tissue from the gut, ovaries, and fat body were used for light and TEM. The tissues were treated with lactophenol blue for light microscopy to stain the fungal cell walls [30]. Calcofluor white was used to detect the chitin [31] and DAPI for visualizing nuclei. For TEM, the fixing solution was added to the insect with the first incision of the dissection procedure and the tissue was fixed with 2% glutaraldehyde for 2 h and 1% osmium tetroxide for 1 h and dehydrated with ethanol. Then, we added propylene oxide and embedded it in LR white resin. Grids were stained with 4% uranyl acetate and 1% lead citrate. We used a Leica microscope with LAS DFC295 software for light microscopy and a Carl Zeiss, Germany LIBRA 120transmission electron microscope.

### 2.4. Fungal Identification

Fungal DNA was obtained from liquid cultures using the Quick-DNA fungal/bacterial miniprep kit by Zymo Research according to the kit protocol. DNA was PCR amplified with ITS1-2 and ITS2 primers, as described [23]. PCR products were sequenced at Macrogen Inc. (Seoul, Republic of Korea) Sequences were uploaded at GenBank with the accession numbers: PP724723-PP724743.

The microscopic fungal structures were observed from 14-day microcultures stained with lactophenol blue with an optical microscope at 40 and 60×. For morphological identification, we used taxonomy guides for fungi [32].

ERIC-PCR patterns were obtained from fungal DNA using ERIC primers [33]. Bands were visualized in agarose gels.

### 2.5. Fungal Isolation from Cacti

To determine if cochineal fungi originated from the cacti on which cochineals feed, we obtained fungal isolates from the cactus leaves used for insect propagation. Cactus leaves were superficially disinfected twice with ethanol at 100% and 70% and then the cuticle was removed under sterile conditions. One cm^3^ of the internal cacti tissue was placed on individual agar plates with the same media to isolate fungi from the cochineals. We also homogenized the inner tissues, prepared three dilutions (10^0^, 10^−1^, and 10^−2^), and dispersed them in culture plates with glass beads. We incubated the plates at 28 °C for 22 days.

### 2.6. Plant-Polysaccharide Degradation in the Cochineal Intestines

Protein gut extracts were obtained from 90 adult female insects, which were dissected, and their intestines homogenized by mechanical disruption. The intestinal tissue particles were separated by centrifugation at 16,000 rpm, and the supernatant was resuspended in a PBS solution with a protease inhibitor (Roche) and sterilized by microfiltration.

The presence of enzymatic activities, including cellulases, amylases, and pectinases, was determined using the dinitrosalicylic acid (DNS) assay to quantify the reducing sugars. To determine the enzymatic activities, 30 µL of gut extract was incubated with each substrate at 2% in 0.1 M phosphate buffer (pH 8). Each reaction was incubated at 28 °C for 24 h, and 50 µL of DNS was added. The reactions were incubated in boiling water for 5 min and transferred to ice for 5 min. Distilled water (0.5 mL) was added, and the absorbance was read at 540 nm using the 0 h time point as a blank. A standard glucose curve (0–2 g/L) was used to calculate the reducing sugar content. With glucose as the standard, we obtained a correlation coefficient of 0.99.

Additionally, we detected simple sugars in the protein gut extract with thin-layer chromatography (TLC) and after incubation with each substrate at 2% for 5 min and 3 h at 30 °C. Glucose and galacturonic acid standards indicated cellulose, starch, and pectin degradation. We placed 20 µL of the gut extract on a silica gel aluminum-backed plate (Merck, Rahway, NJ, USA) and eluted twice with butanol:acetic acid: water (60:30:30 *v*/*v*/*v*) for 12 h, drying between cycles. It was then developed with alpha-naphthol at 100 °C for 2–3 min.

### 2.7. In Planta Fungicide Treatment of Cochineals

We used fludioxonil (Sigma, St. Louis, MA, USA) at 50 µL/mL (180 µM) in 20 µL injections every 5 cm^2^ of the cactus leaf surface. Daily application was performed for seven consecutive days. After treatment, the cochineals did not look damaged and were similar to the control cochineals. We performed the DNS assay to detect polysaccharide degrading activities in the guts of cochineals detached from cacti before and after the fungicide treatment.

### 2.8. Plant-Polysaccharide Degrading Capabilities of Fungal Isolates

For the in vitro qualitative assays, the fungi were placed directly on petri dishes with agar containing each substrate: cellulose 2 g/L, starch 20 g/L, and pectin 20 g/L. Plates were incubated for 24–72 h. Cellulose degrading activity was visualized by adding Congo red 0.02%. Amylase activity was revealed using Lugol (iodine 1% and potassium iodide 2%), and pectinase activity was detected with CTAB (cetyl trimethyl ammonium bromide) 5%. Fungal growth and halos were measured in centimeters and ratios were used as arbitrary units to compare among isolates

## 3. Results

### 3.1. Fungal Isolates from Cochineals

**Figure 1:**
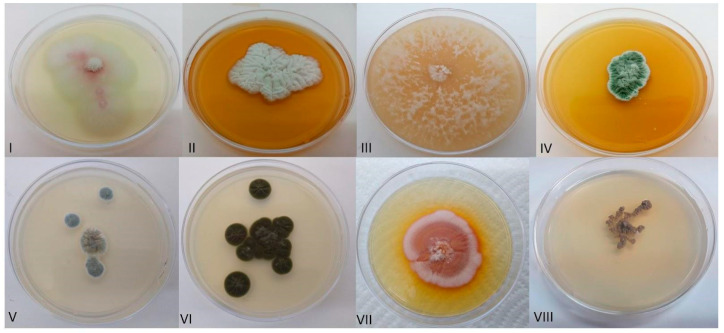
Fungal isolates culture plates belonging to (**I**) *Periconia* sp., (**II**) *Aspergillius hiratsukae*, (**III**) *Arthrinium gutiae*, (**IV**) *Penicillium brevicompactum*, (**V**) *Penicillium olsonii*, (**VI**) *Cladosporium cladosporioides*, (**VII**) *Coniochaeta* sp., and (**VII**) *Microascus verrucosus*.

We sampled only adult female cochineals and not different developmental stages. We obtained 20 morphologically different isolates (Table 1) (Figure 1) from the gut, ovaries, and fat body, corresponding to 12 fungal species (according to the ITS sequence), all belonging to the Ascomycota phylum in six orders: Eurotiales (45% of all isolates), Pleosporales (5%), Microascales (5%), Coniochaetales (5%), Xylariales (10%), and Capnodiales (30%).

### 3.2. Fungi inside the Cochineal

**Figure 2:**
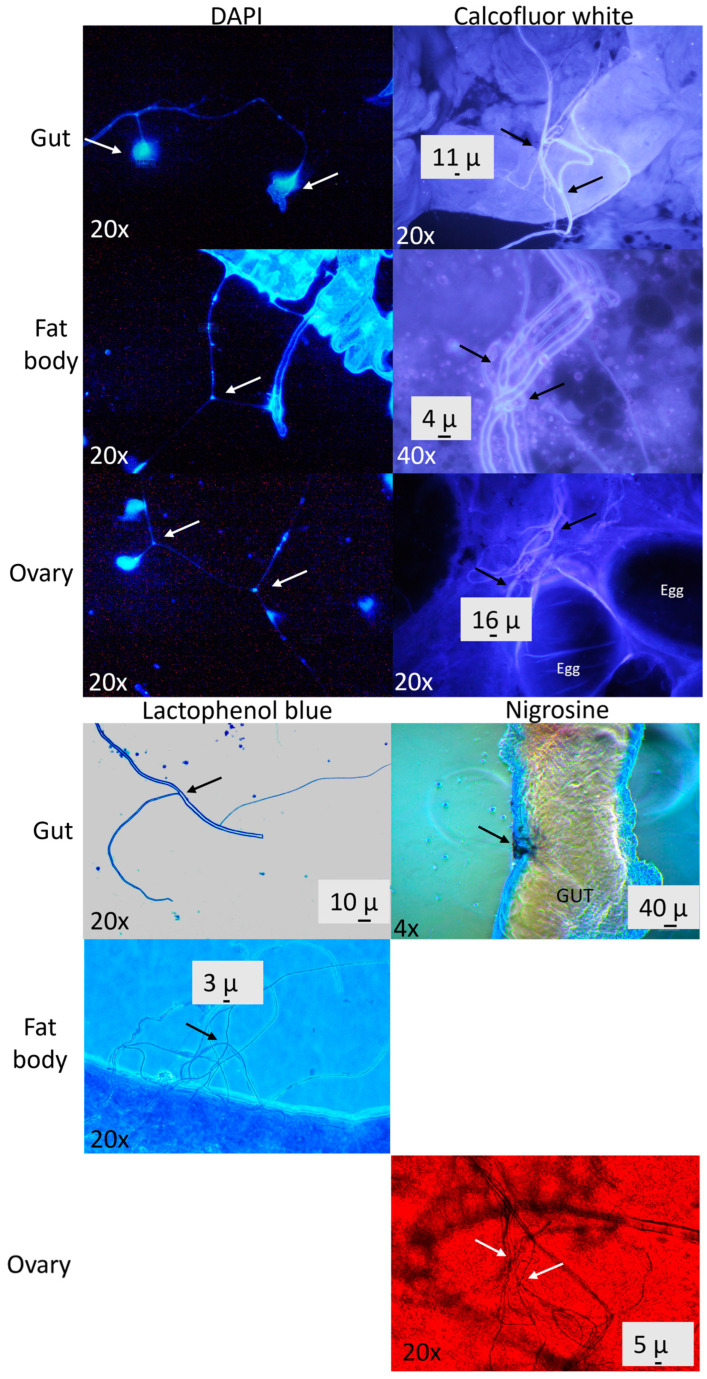
Micrographs of *Dactylopius coccus* tissues: gut, fat body, and ovary, stained with lactophenol blue, calcofluor white, nigrosin, and DAPI, showing hyphae-like structures (indicated with arrows).

We observed some hyphae-like structures in the gut, fat body, and ovaries with light microscopy with blue lactophenol, calcofluor white, and nigrosine staining, and we also found these structures to be DNA-dense with DAPI fluorescence (Figure 2).

### 3.3. Fungal Isolates from Cacti

**Figure 3:**
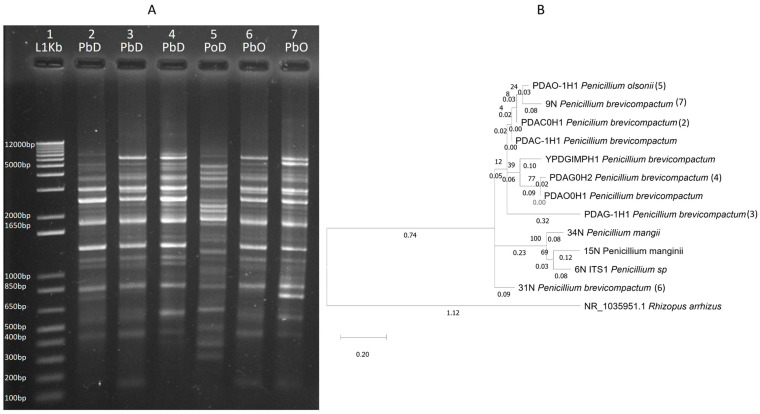
(**A**) ERIC-PCR patterns of *Penicillium* isolates from *Dactylopius coccus* (PbD and PoD) and *Opuntia* (PbO). (1) 1 Kb marker, (2) *P. brevicompactum* PDAC0H1, (3) *P. brevicompactum* PDAG-1H1, (4) *P. brevicompactum* PDAG0H2 from *D. coccus*, (5) *Penicillium olsonni* PDAO-1H1 from *D. coccus*, (6) *P. brevicompactum* 31N, (7) *P. brevicompactum* 9N from *Opuntia* plants. (**B**) Molecular phylogeny of fungal ITS1-2 with Maximum Likelihood and Kimura 2-parameter model with 1,000 bootstraps.

From cactus leaves, we obtained 38 fungal isolates (Supplementary Table S1) (PP892009-PP892046) belonging to eight genera; *Alternaria*, *Cladosporium*, *Purpureocillium*, *Penicillium*, *Aspergillus*, *Mucor*, *Keithomyces*, and *Cordyceps*, three of which are also present in *D. coccus* (*Cladosporium*, *Penicillium*, and *Aspergillus*). A phylogenetic tree of fungal isolates from the cacti and the insect showed that some *Penicillium* isolates from the insect and the cacti were genetically similar (Figure 3). Furthermore, *Penicillium brevicompactum* isolates from cacti and cochineals had identical PCR patterns obtained with ERIC primers (Figure 3). ERIC-PCR fingerprinting has been used in fungal diversity studies [34].

### 3.4. Enzymes for Plant–Polysaccharide Degradation in the Intestines and a Fungicide Treatment in Opuntia Leaves Infected with Cochineals

**Figure 4:**
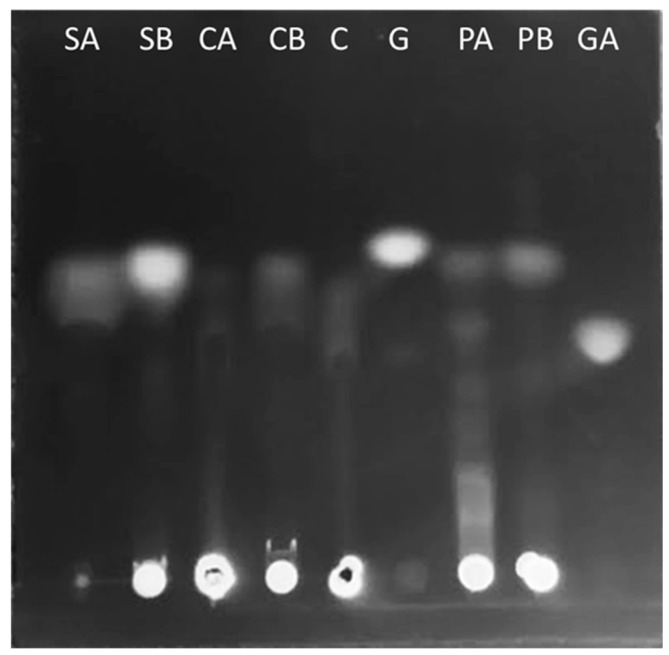
Thin-layer chromatography of sugars produced by the gut extract after 5 min (SA) and 3 h (SB) incubation with starch, after 5 min (CA) and 3 h (CB) incubation with cellulose, and after 5 min (PA) and 3 h (PB) incubation with pectin, compared to gut extract without substrate (C). Glucose (G) and galacturonic acid (GA) were used as standards.

**Figure 5:**
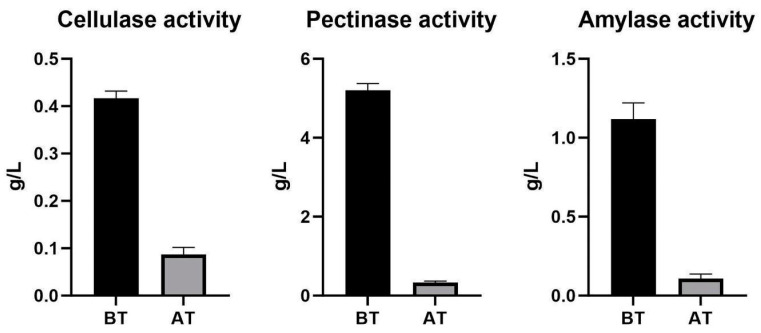
Enzymatic activities of cellulase, pectinase, and amylase before (BT) and after (AT) treatment with fludioxonil fungicide according to DNS assay. Measurements provided as g/L of reducing sugars.

We found cellulase, pectinase, and amylase enzymatic activities in the cochineal gut extracts with the DNS method. Using thin-layer chromatography, we observed an increase in glucose and galacturonic acid after incubation with the gut extracts with cellulose, starch, and pectin confirming the presence of active enzymes in guts (Figure 4). The decreased activities (*p* < 0.5) after the fungicide treatment (Figure 5) were consistent with the hypothesis that associated fungi provide these enzymes in cochineal guts.

### 3.5. Plant-Polysaccharide Degrading Capabilities in Fungal Isolates

Substrates tested for degradation with fungal cultures in vitro were pectin, cellulose, and starch. We used guaiacol for laccases. The values of the ratio of the halo in culture plates, divided by the fungal colony size, in centimeters, were the arbitrary units to compare between isolates. All cochineal fungal isolates showed degrading activities (Table 1). All fungal isolates tested had cellulase activity and all but *Arthrinium* had pectinase activity. All but *Periconia* and *Coniochaeta* had amylase activity. It should be noted that distinct isolates within *Cladosporium* or *Microascus* did not show identical activities. Only *Cladosporium* (with few exceptions) had laccase activity. Laccases can degrade carminic acid.

### 3.6. Culture Conditions and Morphology of Coniochaeta

**Figure 6:**
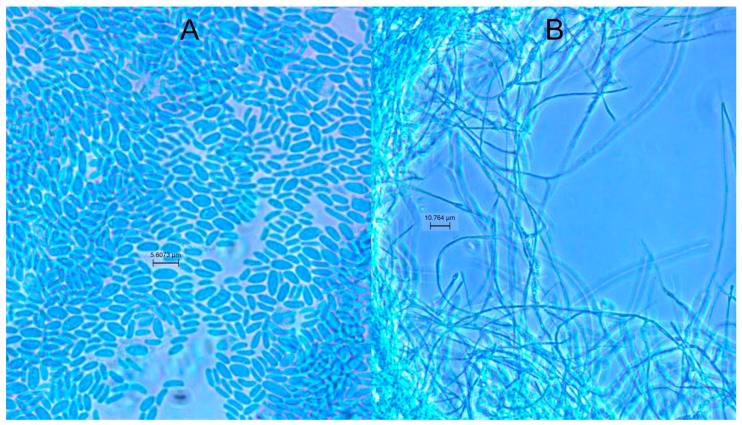
*Coniochaeta* dimorphic morphology (**A**) yeast-like (**B**) filamentous form.

*Coniochaeta* is a dimorphic fungus (Figure 6) and we tested culture conditions that would drive the formation of yeast or hyphal cells. Yeast was predominant in a peptone-yeast medium in aerobic conditions. *Coniochaeta* grew at 37 °C but temperature did not affect the morphology, as observed in most pathogenic dimorphic fungi [35].

## 4. Discussion

### 4.1. On the Origin of Fungi in Cochineals

Insects are frequently colonized by fungal symbionts that may benefit the host [12,36,37]. From cochineal dissected guts we isolated distinct fungi belonging to Ascomycota. Some of the cochineal fungi encountered in their guts may be acquired directly from the cactus leaves where the cochineals grow and feed. Using ITS sequences, we identified *Penicillium*, *Aspergillus*, and *Cladosporium* fungi common to cacti and cochineals. Genomic fingerprints used to characterize bacteria and fungi strains [33] showed identical ERIC-PCR patterns in some *Penicillium* isolates from cacti and cochineals. *Penicillium*, *Cladosporium*, and *Aspergillus* were previously reported in cacti [38].

Besides being in guts, fungi may be encountered inside the cochineal. We detected them during microscopic examinations of the fat body, hemolymph, and ovaries, and the observed structures were detected with dyes used to reveal the fungi. Some of the fungi observed inside the cochineal showed similar fungal structures to those observed in fungal cultures. Insect-associated fungi have been reported in different insect tissues [39,40] in the form of yeast and yeast-like hyphae and spores [41], but there are no reports of hyphae in the ovaries as we found here, which led us to suggest that some fungi may be maternally transmitted besides being obtained from cacti.

### 4.2. Roles of Fungi in Cochineals

In carmine cochineals, we explored the role of fungi in digestion. Our results showed active plant-polymer degrading enzymes in the cochineal guts, which can help degrade the ingested polymers and transform them into assimilable forms. We found that fungi seemingly produced enzymes in the gut such as cellulases, amylases, and pectinases because fludioxonil-treated cochineals barely exhibited those activities in their guts. Fludioxonil was previously used with other fungicides such as ketoconazole and amphotericin B to test the role of fungi in urease production inside the cochineals [29]. Several studies highlight the importance of fungal communities in plant polymer degradation, i.e., the leaf-cutting ant has fungal gardens and fungal communities that are present in the mycetome of some beetles [39,42] or the case of the tortoise leaf beetles (Chrysomelidae: Cassidinae) and their obligate fungal symbiont Stammera, which is indispensable for the degradation of the pectin found in the beetle feed [43]. Our previously published results reveal that animals eat endophytic bacteria [44]. Remarkably, fungi from cacti may be ingested by humans because these cacti are edible, nutrient-rich, and may be eaten raw or juiced for human consumption. Some of these fungi produce mycotoxins [45]. In humans, gut bacteria are responsible for plant-fiber digestion [28], but the role of fungi in digestion has not been fully explored.

Using the cochineal fungal isolates, we detected enzymatic activities in cultures. *Penicillium*, *Cladosporium*, and *Aspergillus* are known to be capable of producing pectinases, cellulases, and xylanases [26]. Fungi are well appreciated in biotechnological applications for their efficient degradation of plant polymers [46,47], and cochineals profit from that.

Notably, from the fat body, we cultured *Periconia* fungal isolates that produced a purple pigment with visual similarities to carminic acid. Certainly, it is worth studying the chemical structure of the novel purple pigment produced by *Periconia.* There are no reports of *Periconia*-producing pigments. *Penicillium, Coniochaeta,* and *Periconia* are known to produce a wide diversity of metabolites and antibiotics [48,49,50], which could help control the cochineal microbial populations if produced inside the cochineal. Antibiotic production by these fungi deserves to be explored.

In culture, we obtained the dimorphic fungus *Coniochaeta* and tested conditions driving its morphological switch, finding that nutrient-rich media in aerobic conditions favor yeast over hyphal growth. Entomopathogenic dimorphic fungi change their cellular morphology from hyphae to yeast inside the insect hemocoel to escape the Toll immune pathway. This morphological switch is driven by nutrient and oxygen concentrations [51].

We found some fungi similar to those previously reported by Vera Ponce de Leon et al. [52], such as *Periconia*, *Penicillium*, *Cladosporium,* and *Aspergillus*, despite the collection sites being separated by kilometers and a time difference between the studies of a few years. Some of the common fungi encountered in both studies may constitute the core fungal community and perhaps the cochineal holobiont (complete organism) [53], together with the nitrogen-fixing *Candidatus* Dactylopiibacterium carminicum, which is a primary symbiont in wild and domesticated cochineals [54].

### 4.3. Perspectives

It has been reported that fungal symbionts in insects produce lipids including sterols [55,56]. In our laboratory, we are exploring whether cochineal fungi produce them. Moreover, since we found that some isolated fungi produce pigments, we have obtained the genome sequence of the *Periconia* sp. fungus and expect that its analysis will reveal the biosynthetic pathway for pigment production. In addition, as it is known that fungi such as *Penicillium* produce antibiotics, our fungal collection may be very valuable because it could be used to obtain novel antibiotics. All this would lead us to propose new strategies to assess the role of fungi in cochineal fitness and to explore the interactions of different fungi among themselves and with the resident bacteria.

## Figures and Tables

**Table 1:** *Dactylopius coccus* fungal isolates and enzymatic activities.

Isolate ID	ITS Identification	% Coverage	% Identity	Tissue	Isolation Medium	Pectinase	Amylase	Cellulase	Laccase
EMC-1H1	*Periconia* sp.	100	99.2	Fat Body	EM	+	-	+	-
PDAC0H1	*Penicillium brevicompactum*	100	99	Fat Body	PDA	+	+	+	-
PDAC-1H1	*Penicillium brevicompactum*	100	99.6	Fat Body	PDA	+	+	+	-
PDAC0H2	*Aspergillius hiratsukae*	99	100	Fat Body	PDA	+	+	+	-
YPDG0H1	*Coniochaeta* sp.	99	99.8	Gut	YPD	+	-	+	-
PDAG-1H1	*Penicillium brevicompactum*	78	100	Gut	PDA	+	+	+	-
YPDGIMPH2	*Cladosporium cladosporioides*	100	99.6	Gut	YPD	+	+	+	+
YPDGIMPH1	*Penicillium brevicompactum*	99	87	Gut	YPD	+	+	+	-
PDAG0H2	*Penicillium brevicompactum*	100	87	Gut	PDA	+	+	+	-
EMGIMPH1	*Cladosporium cladosporioides*	100	98.8	Gut	EM	+	+	+	-
PDAG-1H2	*Cladosporium* sp.	100	99.2	Gut	PDA	+	+	+	+
PDAG0H1	*Cladosporium* sp.	100	100	Gut	PDA	+	+	+	-
PDAH0H1	*Microascus verrucosus*	86	96	Hemolymph	PDA	+	+	+	+
YPDH-1H1	*Arthrinium* sp.	98	98.7	Hemolymph	YPD	-	+	+	-
PDAH-1H1	*Arthrinium* sp.	99	98.5	Hemolymph	PDA	+	+	+	-
YPDH0H1	*Cladosporium sphaerospermum*	100	99.8	Hemolymph	YPD	+	+	+	+
PDAO-1H2	*Cladosporium tenuissimum*	100	100	Ovary	PDA	+	+	+	+
PDAOIMPH1	*Arthrinium gutiae*	99	99.4	Ovary	PDA	-	+	+	-
PDAO0H1	*Penicillium brevicompactum*	100	99.6	Ovary	PDA	+	+	+	-
PDAO-1H1	*Penicillium olsonii*	100	99.6	Ovary	PDA	+	+	+	-

## Data Availability

All relevant data can be found within the manuscript.

## Supplementary Materials

The following supporting information can be downloaded at: https://www.mdpi.com/article/10.3390/insects15100783/s1, Table S1: *Opuntia* fungal isolates.
