# Supplementary material for: On the Origins of Symbiotic Fungi in Carmine Cochineals and Their Function in the Digestion of Plant Polysaccharides
Source: Insects. 2024 Oct 9;15(10):783. doi: 10.3390/insects15100783 (PMC11508352; doi:10.3390/insects15100783)
Supplement: Supplementary file 1 [file insects-15-00783-s001.zip › insects-3085549-supplementary.pdf]

Table S1: *Opuntia* fungal isolates

ID	ITS identification	% identity	% coverage
1N	*Alternaria Tenuissima*	99.8	51
13N	*Aspergillius* sp.	92	89.8
16N	*Aspergillius versicolor*	94.8	57
35N	*Cladosporium cladosporoides*	99	49
12N	*Cladosporium dominicanum*	98.4	100
2N	*Cladosporium* sp.	99.8	99
7N	*Cladosporium* sp.	98.9	48
29N	*Cladosporium* sp.	99	73
5N	*Cladosporium sphaerospermum*	100	43
8N	*Cladosporium sphaerospermum*	96.1	50
10N	*Cladosporium sphaerospermum*	99.6	49
11N	*Cladosporium sphaerospermum*	98.9	47
14N	*Cladosporium sphaerospermum*	99.5	47
23N	*Cladosporium sphaerospermum*	99	49
25N	*Cladosporium sphaerospermum*	100	48
26N	*Cladosporium sphaerospermum*	98	46
27N	*Cladosporium sphaerospermum*	100	100
32N	*Cladosporium sphaerospermum*	100	93
36N	*Cladosporium sphaerospermum*	99	49
37N	*Cladosporium sphaerospermum*	98	44
38N	*Cladosporium sphaerospermum*	100	100
4N	*Cladosporium tenuiisimum*	99.8	51
17N	*Cladosporium uredinicola*	92.7	66
33N	*Cordyceps cateniannulata*	100	99
30N	*Keithomyces carneus*	99	99
20N	*Mucor circinelloides*	88.5	89
21N	*Mucor fragilis*	99	99
22N	*Mucor fragilis*	79	64
24N	*Mucor fragilis*	99	99
28N	*Mucor fragilis*	100	98
18N	*Mucor* sp.	98.6	58
19N	*Mucor* sp.	91.6	60
31N	*Penicilium brevicompactum*	97	61
34N	*Penicilium manginii*	98	52
9N	*Penicillium brevicompactum*	97	74
15N	*Penicillium manginii*	98.8	51
6N	*Penicillium* sp.	96.8	42
3N	*Purpureocillium* sp.	99.9	100
